# Endothelialization of PTFE-covered stents for aneurysms and arteriovenous fistulas created in canine carotid arteries

**DOI:** 10.1038/s41598-024-55532-5

**Published:** 2024-02-27

**Authors:** Lei Yang, Xiaohong Hao, Bulang Gao, Chunfeng Ren, Hong Du, XianHui Su, Dongliang Zhang, Tong Bao, Zongrong Qiao, Qinying Cao

**Affiliations:** Shijiazhuang People’s Hospital, Shijiazhuang, 050011 Hebei Province People’s Republic of China

**Keywords:** Covered stent, Bare stent, Aneurysm, Arteriovenous fistula, Endothelialization, Diseases, Medical research, Neurology

## Abstract

To investigate the endothelialization of covered and bare stents deployed in the canine carotid arteries and subclavian arteries for treating experimental aneurysms and arteriovenous fistulas, twenty aneurysms were created in 10 dogs, and 20 fistulas in another 10 dogs. The Willis balloon-expandable covered stent and a self-expandable covered stent were used to treat these lesions, and a self-expandable bare stent was deployed in the subclavian artery for comparison. Followed up for up to 12 months, the gross observation, pathological staining, and scanning electronic microscopic data were analyzed. Two weeks after creation of animal model, thirty self-expandable covered stents and ten balloon-expandable covered stents were deployed. Fifteen bare stents were deployed within the left subclavian arteries. Twenty days after stenting, the aneurysm significantly shrank. At 6 months, the thrombi within the aneurysm cavity were organized. Three to 12 months later, most covered and bare stents were covered by a thin transparent or white layer of endothelial intima. Layers of intima or pseudomembrane were formed on the stent 20–40 days after stent deployment. Over three months, the pseudomembrane became organized, thinner, and merged into the vascular wall. Under scanning electronic microscopy, the surface of covered and bare stents had only deposition of collagen fibers and rare endothelial cells 20–40 days after stenting. From three to ten months, the endothelial cells on the internal surface of stent became mature, with spindle, stripe-like or quasi round morphology along the blood flow direction. Over time, the endothelial cells became mature. In conclusion, three months after deployment in canines’ arteries, the self-expandable bare and covered stents have mostly been covered by endothelial cells which become maturer over time, whereas the balloon-expandable covered stents do not have complete coverage of endothelial cells at three months, especially for protruding stent struts and areas. Over time, the endothelialization will become mature.

## Introduction

Cerebral vascular diseases like intracranial aneurysms and arteriovenous fistulas may severely damage the health of patients. Cerebral aneurysms are the most common reason for non-traumatic subarchnoid hemorrhage, and unruptured ones have a high incidence of approximately 7% in community residents aged 35–75 years^1–3^. Arteriovenous fistulas are abnormal communications between arteries and veins difficult to manage^4,5^ and may produce some significant pathophysiological changes in the cardiovascular system, including changing the systemic hemodynamic status, cardiac loads, and levels of vasoconstrictors and vasodilators. These effects may contribute to great morbidity and mortality, especially in the presence of high-flow fistula. Surgical treatment of these diseases may carry a high risk of periprocedural complications and mortality to say nothing of patients who may not be able to tolerate the surgery. Endovascular therapy has become acceptable for poor surgical candidates or cases with difficulty of management, especially use of covered stents. Covered stents have been used to treat intracranial and extracranial carotid aneurysms, carotid-cavernous fistulas, pseudoaneurysms, and recurrent and blister aneurysms, resulting in good effects^6–14^. The use of covered stents has made it possible to operate only within the parent artery without navigating endovascular devices into the aneurysm cavity or fistula for embolization. This approach has greatly decreased the possibility of intraprocedural rupture of aneurysms or arteries, increased aneurysm occlusion rate, simplified endovascular operation, and reduced periprocedural complications. Nonetheless, long-term application of antiplatelet therapy is needed after deployment of the stents, because the metallic stents and materials like polytetrafluoroethylene (PTFE) used to make the covered stents are foreign bodies and may produce thrombi to occlude distal vessels, leading to severe consequences. Complete endothelialization with endothelia cells covering the whole stent will embed the stent within the arterial wall and isolate the foreign materials of stent from blood flow, and thus, the covered stent will become a part of the vessel itself, without producing bad effects of thrombogenesis. However, endothelialization of these stents has been rarely investigated in animal experiments, and the time for endothelial cells to completely cover the entire stent is unknown neither in animals nor in humans. Aneurysms and arteriovenous fistulas have been created in animals for relevant studies of endovascular treatment^4,5,15–19^. It was thus hypothesized that the endothelialization of covered stents could be tested in animal experiments. This study was consequently performed to investigate the endothelialization of covered stents in animal experiments, in which aneurysms and arteriovenous fistulas were created in animal (canine) carotid arteries followed by deployment of covered stents for investigating the endothelialization of covered stents.

## Materials and methods

### Animals

This study was approved by the Ethics Committee of Shijiazhuang People’s Hospital, and the guidelines of animal care had been followed. All methods were performed in accordance with the relevant guidelines and regulations, and the study was reported in accordance with ARRIVE guidelines. Twenty 1-year-old greyhound dogs of either sex with a weight of 20–25 kg were used for the experiment. Twenty aneurysms were constructed in 10 dogs, and 20 arteriovenous fistulas in another 10 dogs. All dogs had a standard experimental diet. After overnight fasting, thiopental 15–20 mg/kg was used for sedation of the dogs. After endotracheal intubation, isoflurane 1%-3% was used for continuous sedation and anesthesia via the endotracheal tube. The dog neck was prepared and sterilized, and the blood pressure, heart rate, oxygen saturation, electrocardiogram and depth of anesthesia were monitored during the experiment.

### Construction of aneurysm and arteriovenous fistula models

**Figure 1:**
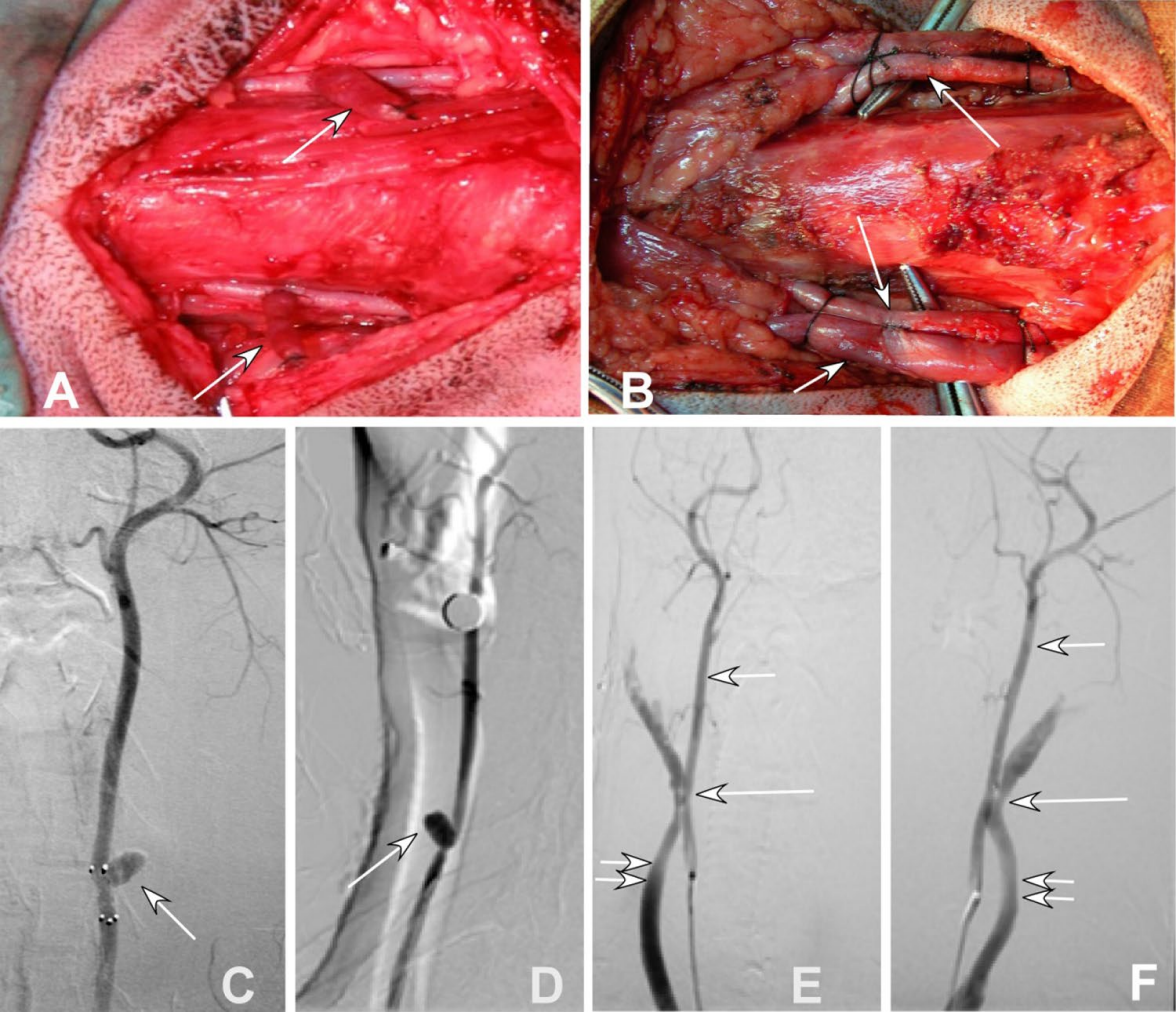
Construction and angiography were shown of carotid lateral-wall aneurysm and Jugular-vein-carotid artery fistula of canine. (**A**) Two aneurysms (arrows) were constructed with a venous pouch from two segments of jugular vein being sutured onto the lateral wall of the carotid artery of dogs. (**B**) Two fistulas between the jugular vein and the carotid artery were constructed with the long arrow indicating the carotid artery and the fistula and the short arrow indicating the jugular vein and the fistula. C&D. Angiography of the aneurysms (**C** anteroposterior position and **D** lateral position) was shown, with the arrow indicating one aneurysm. E&F. Angiography of the fistula between the jugular vein and carotid artery on the right (**E**) and left (**F**) was performed, with two small arrows indicating the jugular vein, a small arrow indicating the carotid artery, and one long arrow pointing to the fistula.

Aneurysm construction was performed with similar skills used in other studies^15–17,20^. Briefly, after a midline incision of 10 cm was made in the neck, the left external jugular vein (EJV) was exposed for 6 cm long. A 3-cm long segment of the EJV was excised to produce a venous pouch. After the left common carotid artery (CCA) was exposed, an arteriotomy of 4-mm was made on the lateral CCA wall after temporary clamping of the artery. The open end of the EJV pouch was sutured end-to-end to the arteriotomy of the left CCA, forming a berry-shaped side-wall aneurysm model. An aneurysm on the other side was constructed with similar skills. Totally, 20 aneurysm models were constructed in 10 dogs (Fig. 1A).

An arteriovenous fistula was constructed with the skills used in the study by Geremia et al.^18^. In brief, after the EJV and CCA were exposed for a certain length on one same side of the neck, the EJV and CCA were put together side by side for temporary clamping at the proximal and distal ends. After a 2–4 mm incision on the external lateral wall of CCA and internal lateral wall of EJV, the two vessels were sutured together at the incision to form an arteriovenous fistula between the EJV and the carotid artery. A similar arteriovenous fistula was constructed on the other neck side, with a total of 20 fistulas being constructed in 10 dogs (Fig. 1B).

### Covered stents

The Willis balloon-expandable covered stent (MicroPort, Shanghai) and a house-made self-expandable covered stent (MicroPort, Shanghai) were used to treat the experimentally-induced lesions. The Willis stent is composed of three parts: expandable PTFE (ePTFE) membrane, a bare cobalt-chrome stent, and a balloon catheter^6,9,21^. The self-expandable covered stent was made from ePTFE membrane, a bare nitinol stent, and a microcatheter for delivery. The self-expandable bare stent to be deployed in the left subclavian artery for comparison was made of nitinol. The ePTFE covered the external surface of the stent.

### Angiography and deployment of stents

**Figure 2:**
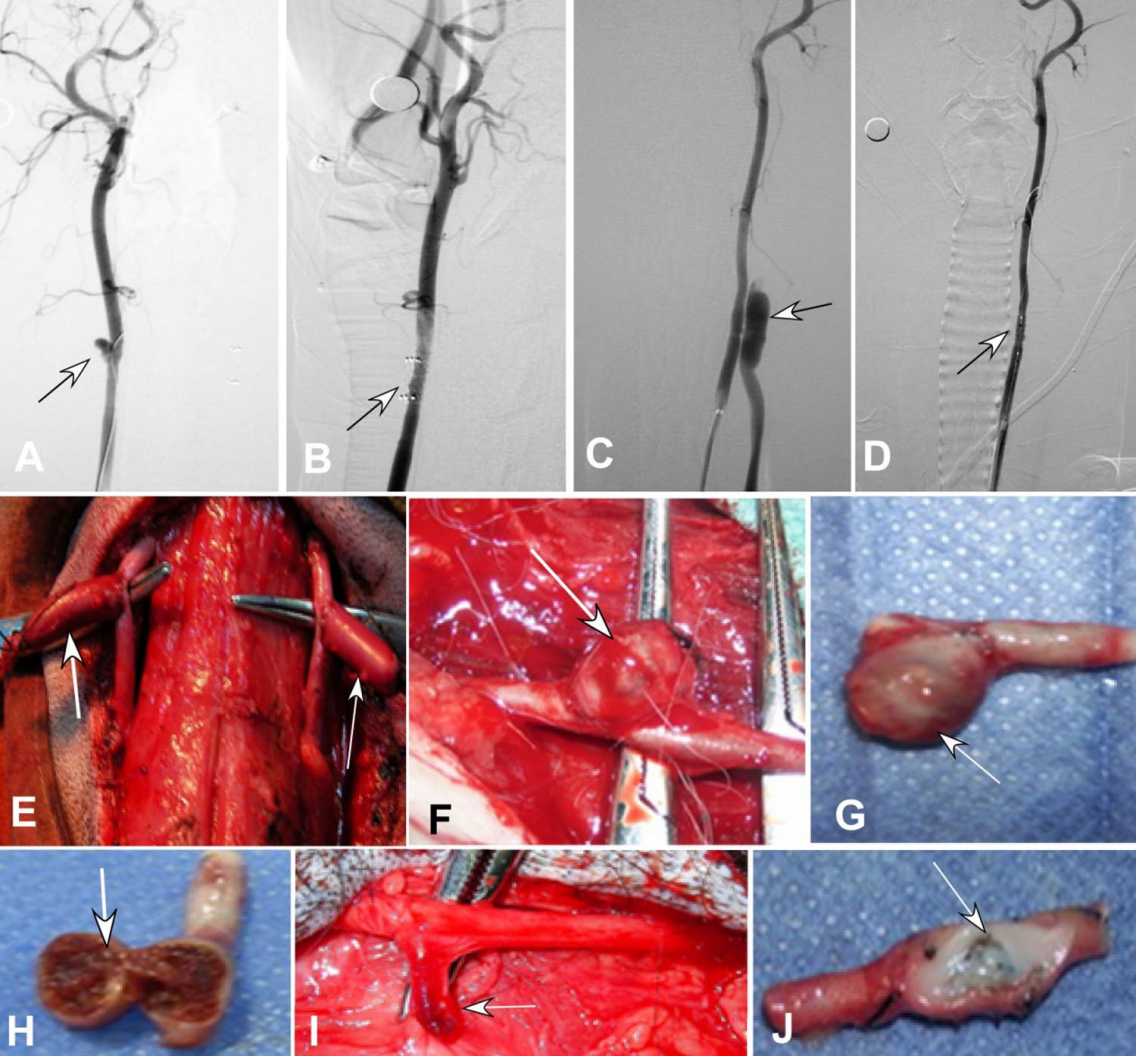
Deployment of covered stents to treat and gross specimen of experimental aneurysms and fistulas. (**A**, **B**). An aneurysm (arrow) made of venous pouches was shown on angiography (**A**), and deployment of a self-expandable covered stent eliminated the aneurysm (**B**). C&D. An arteriovenous fistula (arrow) between the external jugular vein and the carotid artery was shown (**C**), and deployment of the Willis stent immediately occluded the fistula (**D**, arrow). (**E**, **F**) Two aneurysms were made of venous pouches were shown after construction (**E**), and twenty days after deployment of a covered stent, the aneurysm (arrow) shrank significantly in vivo (**F**). (**G**, **H**) The aneurysm was excised (**G**), and non-completely-organized thrombi were presented within the aneurysm cavity after the aneurysm was cut open (**H**, arrow). (**I**) At 6 months, the aneurysm in vivo shrank significantly (arrow). (**J)** The orifice of a fistula between the external jugular vein and the carotid artery was covered by a thin layer of transparent substance (intima).

Two weeks after successful construction of animal models, angiography was performed (Fig. 1C–F) and covered stents were deployed to treat the lesion (Fig. 2A–H). In the first group with seven dogs of aneurysms and three dogs of arteriorvenous fistulas, a self-expandable covered stent was used in one CCA, and a balloon-expandable Willis stent on the other CCA in each dog. In the second group with three dogs of aneurysms and seven dogs of fistulas, a self-expandable covered stent was deployed in each CCA. A self-expandable bare stent was deployed in the subclavian artery in two groups. The components of the animal models (aneurysms or arteriovenous fistulas) were not significantly (P = 0.47) different in group one compared with group two. The stents deployed in each dog were not significantly (P = 0.52) different in group one compared with group two.

### Angiographic evaluation of the lesion

**Figure 3:**
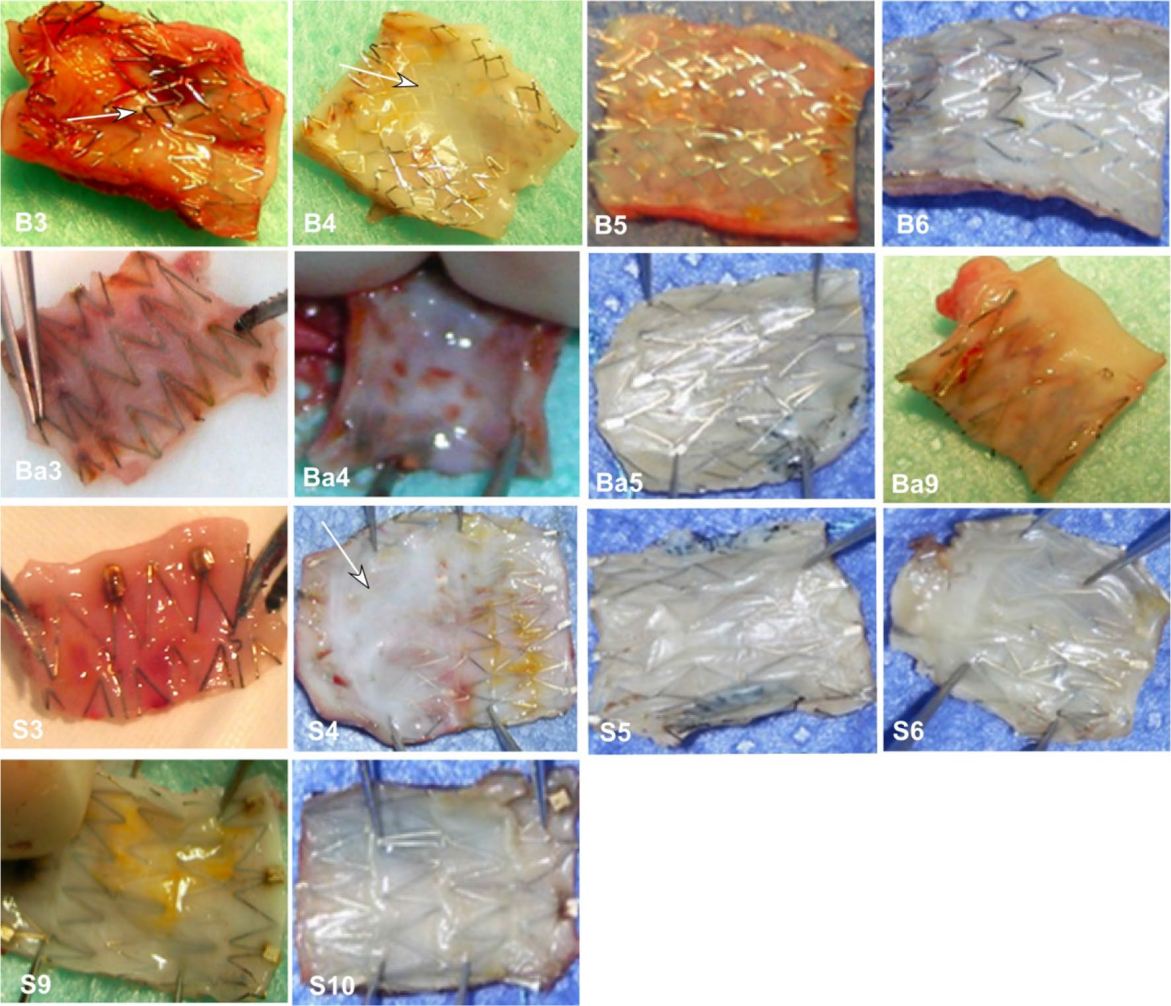
Intima on the inner wall of the stents on gross examination. B3-B6: The intima on the internal wall of the balloon-expandable stents in the carotid artery was shown at 3, 4, 5, and 6 months after stenting, respectively. A thin layer of endothelial cells and intima was shown at all time points, and no intima was shown on stent struts which protruded into the lumen (arrow at 3 months, B3). At depression areas, the intima was thicker (4 months in B4, arrow). Ba3-Ba9: The intima on the internal wall of the self-expandable bare stents which were deployed into the subclavian artery at 3, 4, 5, and 9 months, respectively. The stent struts were expanded well without protrusion in to the lumen. Good intima covered the internal wall of the stent. S3-S10: The intima on the internal wall of self-expandable stents in the carotid artery was demonstrated 3, 4, 5, 6, 9, and 10 months after stenting, respectively. The stent struts were well expanded without protrusion into the lumen. The intima well covered the stent struts with thicker intima at depression areas (4 months in S4, arrow).

**Figure 4:**
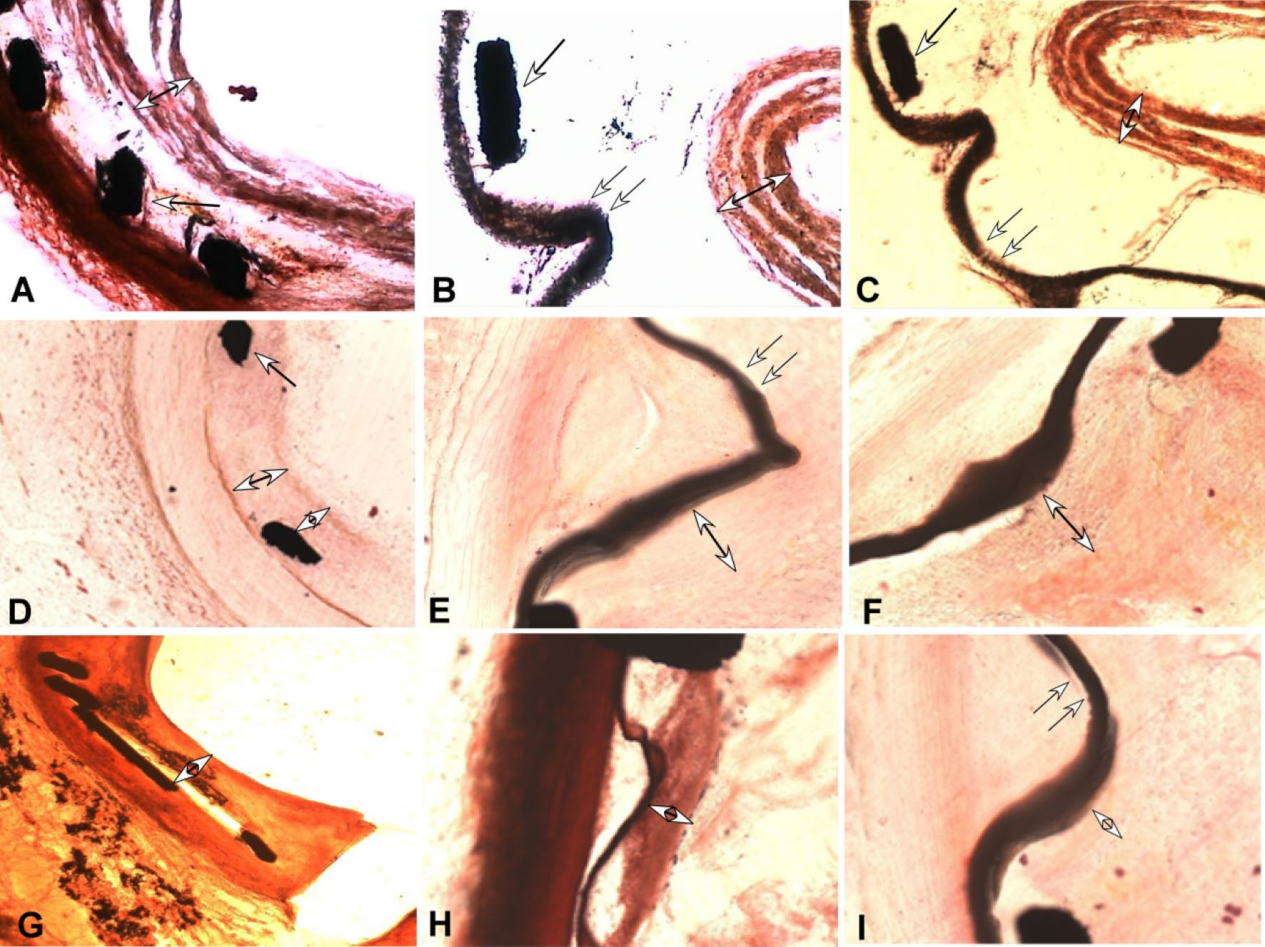
Intima hyperplasia within the stents under optical microscopy. (**A**-**C**) At twenty days, a layer of intima hyperplasia of self-expandable bare stent (**A**), balloon-expandable (**B**) and self-expandable (**C**) stent was shown. At this time, an inner membrane or false membrane was observed in all three types of stent. This membrane was loosely attached to the internal wall of the stent and could be easily shedded into the lumen. (**D**-**F**). At three months after stenting, the layer of intima hyperplasia of the self-expandable bare stent (**D**), balloon-expandable (**E**) and self-expandable (**F**) stent had become organized and thinned and merged into the stented segment of vessel as a whole. (**G**-**I**). At 5 months, the layer of intima hyperplasia of self-expandable bare stent (**G**), balloon-expandable (**H**) and self-expandable (**I**) stent had similar but thinner presentations with that at 3 months. Double arrows indicate the expandable polytetrafluoroethylene (ePTFE) membrane graft covering the stent, an arrow with double heads indicates the thickness of the intima hyperplasia, and a single arrow suggests the stent strut. × 200 times.

**Figure 5:**
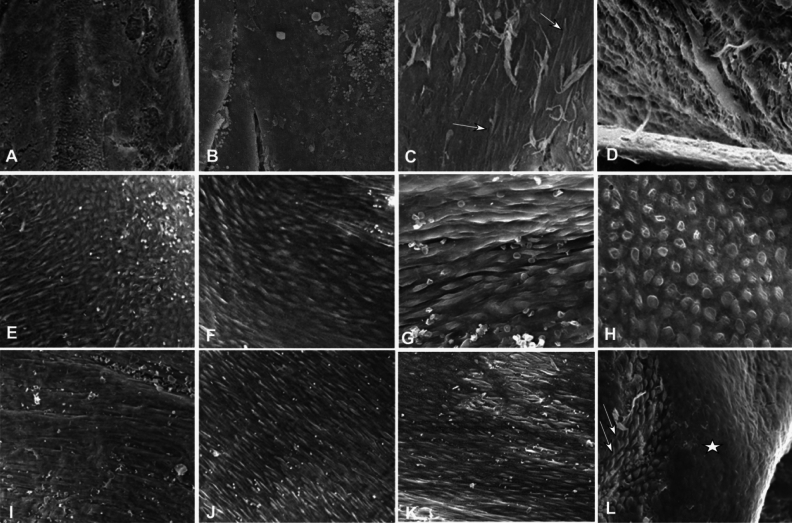
Intima hyperplasia of stents under scanning electronic microscopy was shown. (**A**, **B**) Twenty days after stenting, only some collagenous fibers and red blood cells were shown on the self-expandable covered stent (**A**) and self-expandable bare stent (**B**), with no endothelial cells. (**C**, **D**) Forty days later, more collagenous fibers and red blood cells were presented on the internal surface of the self-expandable bare stent (**C**) and self-expandable covered stent (**D**), with a few stripe-like endothelial cells (arrows in figure **C**). (**E**–**H**) Three months after stenting, fusiform, round and stripe-like endothelial cells were presented at the internal surface of the balloon-expandable covered (**E**), self-expandable covered (F), and self-expandable bare (**G**, **H**) stent. (**I**-**L**) At the 4th month, stripe-like and fusiform endothelial cells were presented on the balloon-expandable covered (**I**), self-expandable covered (**J**), and bare (**K**, **L**) stent. The endothelial cells were fusiform at depressed areas (double arrows in figure **D**) but round at protruding areas (star in Figure **L**).

**Figure 6:**
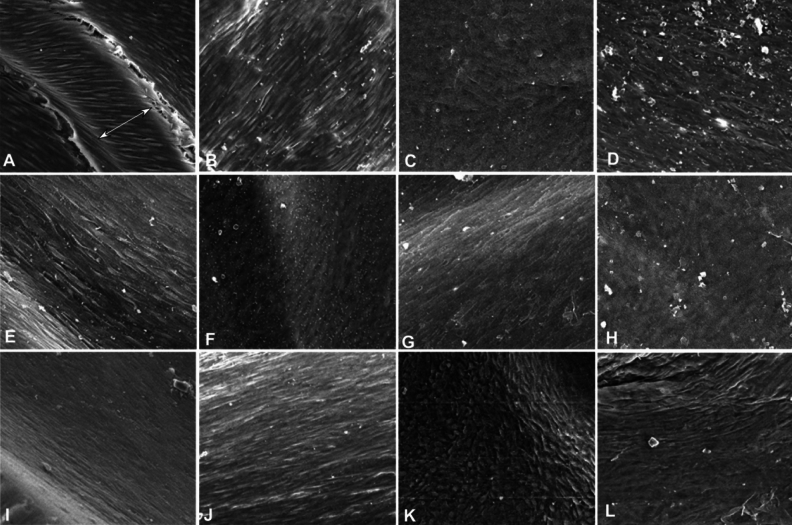
Intima hyperplasia of stents under scanning electronic microscopy was shown 6–10 months after stenting. (**A**-**D**). Five months after stenting, fusiform and stripe-like endothelial cells were presented on the internal surface of balloon-expandable covered (**A**), self-expandable covered (**B**, **C**), and bare (**D**) stent. The arrow in figure A indicates the stent strut. Some materials from the blood were present on the surface. (**E**–**H**). At the 6th month, fusiform and stripe-like endothelial cells were presented on the surface of balloon- (**E**, **F**) and self-expandable (**G**, **H**) covered stent. (**I**-**K**). Nine months later, fusiform and stripe-like endothelial cells were shown on the internal wall of the balloon- (**I**) and self-expandable (**J**) covered stent and bare (**K**) stent. (**L**) Ten months later, fusiform and stripe-like endothelial cells were on the self-expandable covered stent.

**Figure 7:**
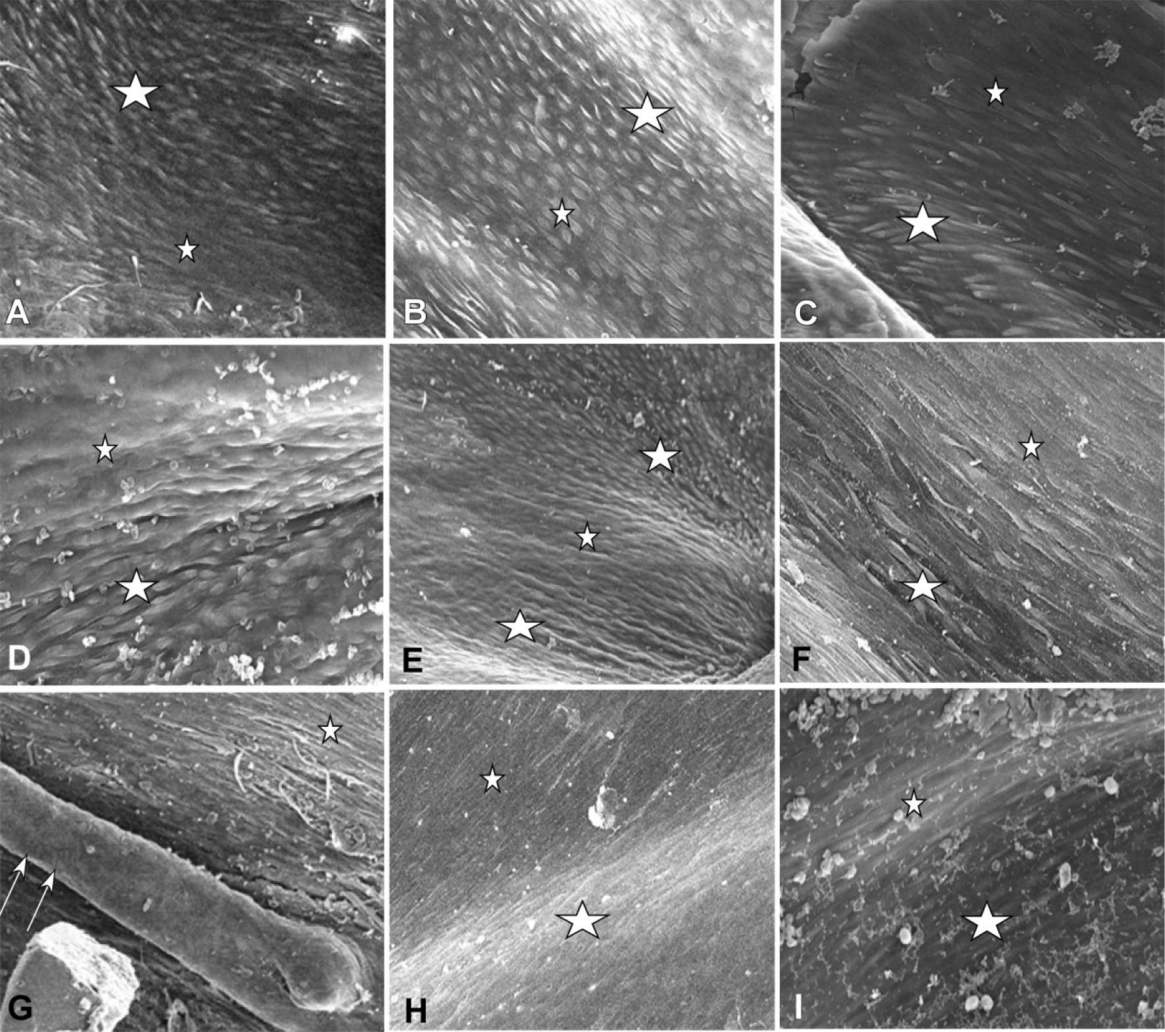
Surface factors may affect the endothelialization of the stents under scanning electronic microscopy. (**A**-**C**) The endothelial cells were morphologically different at flat areas (small stars) from that at protuberance areas (bigger stars) three months after stenting for self-expandable covered stent (**A**), bare stent (**B**), and balloon-expandable covered stent (**C**). The endothelial cells at the flat area were more mature. (**A**, **B**) indicate three months and (**C**) for six months. (**D**-**F**) The endothelial cells were morphologically different at flat areas (small stars) from that at depression areas (bigger star) three months after stenting for self-expandable bare stent (**D** at 3 months and **E** at months) and balloon-expandable covered stent (**F** at 6 months). (**G**) At four-month follow-up, a strut (double arrows) of a balloon-expandable covered stent protruding into the vascular lumen had no endothelial cells on the strut surface, whereas some fusiform and stripe-like endothelial cells were present at the flat area (small star). (**H**, **I)** At six months, the endothelial cells at flat areas (small stars) were similar to those at protruding areas (bigger stars).

Immediately after stent deployment and at the end of model-construction procedure, angiography was conducted to check if any acute thrombosis occurred. Twenty days, 3, 4, 5, 6, 9, 10, and 12 months later, angiography was carried out to observe the vascular patency before sacrifice of the dogs. After sacrifice, the stents and the vessels were harvested for gross observation (Figs.2, 3), pathological staining (Fig. 4), and scanning electronic microscopy to evaluate endothelialization and degree of endothelial maturation over the entire stent surface (Figs. 5–7).

### Statistical analysis

The SPSS software version 12.0 (IBM, Chicago, IL, USA) was used to do the statistical analysis. Continuous data were presented as mean ± standard deviation if they were in normal distribution and tested with the student t test or median and interquartile range if they were not in normal distribution and tested with the Mann Whitney U test. Enumeration data were expressed as numbers and percentage and tested with the Chi square test. The statistically significant P value was set at P < 0.05.

## Results

### Stents deployed

Thirty self-expandable stents were deployed to cover 13 aneurysm models and 17 fistula models (Fig. 1), and ten balloon-expandable stents were used to cover 7 aneurysm and 3 fistula models (Fig. 2). Eight bare stents were deployed within the left subclavian artery in group one and seven bare stents were deployed within the left subclavian artery in group two.

### Gross observation of stent samples

Twenty days after deployment of stents, the aneurysm significantly shrank with non-completely organized thrombi within the aneurysm cavity (Fig. 2). At 6 months, the thrombi within the aneurysm cavity were further organized, and the aneurysm was significantly shrunk. For arteriovenous fistulas, the fistula orifice was covered by a thin layer of transparent substance or endothelial intima (Fig. 2J).

Three to 12 months later, most of the covered stents and bare stents were covered by a thin transparent or white layer of endothelial intima (Fig. 3). In some stents, the stent struts did not attach firmly to the vascular wall and protruded into the vascular lumen, with no intima or only a very thin layer of intima covering the protruded stent struts.

### Observation under optical microscopy

Under optical microscopy (Fig. 4), layers of intima or pseudomembrane were formed on the stent twenty to 40 days after stent deployment. The intima was not firmly attached to the inner wall of stent and could be easily detached at 20 days. At 40 days, the stent intima adhered closely to the vascular wall. Over three months, the pseudomembrane or intima became organized, thinner, and merged into the vascular wall.

### Observation under scanning electronic microscopy

Under scanning electronic microscopy (Figs. 5, 6, 7), the surface of covered and bare stents had only deposition of some collagen fibers, red blood cells but rare endothelial cells, with no marked endothelialization 20–40 days after stenting.

At three months (Fig. 5), little difference was found in the degree of endothelialization among self- and balloon-expandable stents or bare stents, and most of the endothelial cells on the stents were immature and arranged along the direction of blood flow. The shape of endothelial cells was spindle, long stripes, or quasi circles. The extent of stent endothlialization was approximately 20% in the self-expandable stent and 24% in the balloon-expandable stent.

At four months (Fig. 5), the endothelialization of stents was more mature than that at three months. The endothelial cells on the stents became longer, with the long axes of cells being mostly arranged along the direction of blood flow. The boundary between cells became more unclear, the endothelial cells became closely connected with each other, and the shape of cells could not be clearly distinguished. The endothelial cells prostrated on the inner vascular wall, and the surface was more flat and smooth. Part of the metal struts of balloon-expandable stents protruded into the vascular lumen without endothelialization. Stent endothelialization was finished in approximately 65% in the self-expandable stent and 60% in the balloon-expandable stent.

At five months (Fig. 6), the endothelialization was similar to that at four months in all stents. Some common morphological characteristics existed in the endothelial cells, with longer shapes, long cell axes along blood flow direction, unclear boundary between cells, and close connection between cells. Approximately 75% areas of both stents were covered by endothelialization.

At six months (Fig. 6), similar endothelialization existed in both self- and balloon-expandable covered stents, with the cells becoming flat, longer, closely connected, and unclear in boundary. No samples were obtained for bare stents. The stent endothelialization was approximately 90% in the self-expandable stent and 94% in the balloon-expandable stent.

At nine months (Fig. 6), the endothelialization was similar on all stents, with endothelial cells becoming even longer, unclear in boundary, closely connected with each other, and flat on the surface. In relatively bulging areas for self-expandable covered stents, the cells were long, spindle and quasi circular, which obviously protruded on the surface, with clear boundary between cells and the long axes of cells being arranged along the blood flow direction, which may reflect late endothelialization and mild maturation. In relatively flat places, the cells were wide and flat in shape, closely connected with each other, and flat on the surface. Endothelialization was 100% on both stents.

At ten months (Fig. 6), the endothelial cells were relatively flat and closely connected with each other, with unclear boundaries and long axes basically arranged in the blood flow direction. No data were obtained for the balloon-expandable covered stent at ten months or for self-expandable covered stents at 12 months. Endothelialization was found to cover the self-expandable stent (100%). No significant (P = 0.73) difference existed in the stent endothelialization extent at different time points of observation between the self- and balloon-expandable stents.

### Factors affecting stent endothelialization

The endothelialization of stents was different at the flat areas as compared with that at the protuberance or depression areas (Fig. 7). At the protuberance and depression areas, the endothelial cells were relatively immature, characterized by clear outline, obvious boundary and most spindle, quasi round, and small in size. Endothelial cells at the flat area were relatively flat, wide, indistinct, creeping on the intima, with unclear boundaries and tight connections, showing relatively mature. The difference of cell morphology between the flat and protuberance or depression areas may be caused by different blood flow velocities. The blood flow at the flat area was slow, and the cells were easy to adhere and mature; however, the blood flow at the protuberance or depression areas was more rapid, and the cells were not easy to adhere and mature.

If a stent strut protruded into the vascular lumen, no endothelial cells were observed on the strut even after four months (Fig. 7G). The length of observation time also affected the endothelialization: with time, the morphology of endothelial cells at the flat and protuberance or depression areas became more similar and mature.

## Discussion

In this study investigating the endothelializtion of balloon- and self-expandable covered stents and bare stents which were deployed in the canine carotid and subclavian arteries the treatment of experimental aneurysms and arteriovenous fistulas, it was found that three months after deployment in canines’ arteries, the self-expandable bare and covered stents have mostly been covered by endothelial cells which become maturer over time, whereas the balloon-expandable covered stents do not have complete coverage of endothelial cells at three months, especially for protruding stent struts and areas. Over time, the endothelialization will become mature.

After endovascular treatment of vascular lesions like aneurysms, progressive thrombosis of the aneurysm plays an important role in the healing process. In our study, the aneurysm shrank 20 days after deployment of a covered stent, with presence of non-completely organized thrombi within the aneurysm cavity. Six months later, the aneurysm substantially shrank probably after organization of the thrombi within the aneurysm cavity.

The deployment of a metal stent in the artery will denude the endothelial layer of the parent artery^22^. Reendothelialization of the parent artery occurs by seven days in rabbit models, and device endothelialization takes place in a progressive manner over weeks to months. The endothelial cells covering the flow diverters appeared to come from adjacent parental arteries^23,24^, and cell migration from the adjacent parent artery to the stent allows for continuous endothelial lining along the lumen of the parent artery, greatly contributing to complete healing of vascular lesions like aneurysms and fistulas. In the left subclavian artery in our study, a self-expandable nitinol stent was deployed for endothelialization as compared with that of covered stents. The nitinol stent has the advantages in vascular lumen of ease of delivery, high and reliable expansion with a higher expanded diameter compared to the constrained diameter within the microcatheter^25^. The self-expandable ability of the nitinol stent eliminates the requirement of a balloon catheter for deployment, thus reducing the risk of intimal injury and subsequent platelet aggregation and intimal hyperplasia. The self-expandability of the nitinol stent indicates high elasticity compared with the balloon-expandable cobalt-chrome stent which possesses plastic deformation and may recoil. Stent recoiling of balloon-expandable stents will increase the amount of thrombi formation and subsequent arterial stenosis in the stented segment of vessel because of neointimal hyperplasia^26^, and balloon-expandable stents will retain more intimal proliferative reactions than self-expandable stents in swine iliac arteries^27^. A small metal-to-tissue ratio seems to play a significant role in preventing excessive neointimal hyperplasia because of decreased metal component as a foreign material.

However, an ePTFE membrane on the external surface of a stent will significantly increase the component proportion of foreign body and consequently induce a great deal of cellular reaction to the covered stent. In our study, 20–40 days after deployment of a covered stent in the artery, layers of neointima or pseudomembrane were formed on the internal surface of the covered stent, and these layers of pseudomembrane were to isolate the stent foreign materials from the blood flow and could be easily detached from the stent surface. Under scanning electronic microscopy, these layers of pseudomembrane were composed mostly of collagen fibers and red blood cells but rare endothelial cells. Three months after deployment of the covered stent, these layers of pseudomembrane became organized, thinned and merged into the arterial wall, and endothelial cells covered most of the covered and bare stents. But, protruding stent struts and areas of the balloon-expandable stents were still devoid of endothelial cells at 3–4 months after stenting. Therefore, early withdrawing of the dual antiplatelet therapy with aspirin and clopidogrel before complete endothelialization of the covered stents would result in formation of thrombi and possible thromboembolic complications like in-stent thrombosis and distal embolism, leading to ischemic stroke^28^.

In the study investigating in situ tissue engineering to reveal the endothelial growth patterns for flow diverter design^28^, it was found that at locations 5–10 mm to the aneurysm neck and at 60 days, the flow diverters were completely covered with tissue and advanced endothelialization. In other studies of animal models in rabbits^23,29^, complete endothelialization of flow diverters was reported to be present as early as seven days after stent deployment in normal arterial segments and 4–8 weeks after deployment across the aneurysm neck. This fast endothelialization is probably caused by fewer foreign materials in the flow diverters, whereas in the covered stents, a piece of ePTFE membrane covers the whole metal stent to form the covered stent. The amount of foreign materials will induced a greater reaction of the body, requiring a significantly longer time for endothelialization. In a conventional surgical animal (canine) study with interpositional ePTFE graft, both the ePTFE ends of 1–1.5 cm anchoring to the aorta were endothelialized, but the rest ePTFE in the stent middle portion was not^30^. Researchers also showed that if the stent graft was in close contact with arterial wall, the stent graft would be completely covered by glossy white intima with a layer of endothelial cells even at 2-month follow-up^31^. Nonetheless, at an aneurysm segment, the stent graft was covered by brownish thrombi without any endothelial cells 6 months after stenting. ePTFE is the most popular synthetic alternative to autologous vein used as important implants for reconstruction of diseased small- to medium-sized arteries because it produces very little tissue or blood reaction^30^. Thus, ePTFE was used as the stent graft in our study.

Whether the internal surface of the covered stent after deployment was smooth or not affected the endothelialization of the covered stent. The endothelialization at flat and smooth areas was better than that at protuberance or depression areas. The endothelial cells at the flat and smooth areas were maturer, with the cells being relatively flat, wide, indistinct, and of unclear boundaries and tight connection, whereas the endothelial cells at the protuberance and depression areas were relatively immature, characterized by clear outline, obvious boundary, and mostly spindle and quasi round. When deploying the balloon-expandable covered stent, the balloon should be inflated to evenly dilate the stent so that it would expand evenly without marked protuberance or depression on the stent internal surface.

The self-expandable covered stents would continuously expand after deployment in vessels, resulting in better mergence of the stent into the vascular wall. However, the balloon-expandable stents may recoil after complete expansion on deployment, leading to partial protrusion of the stent struts into the vascular lumen. The protruded stent struts had difficulty in endothelialization even four months after deployment as demonstrated in our study. Better design and deployment skills are needed for balloon-expandable covered stents to facilitate endothelialization.

To promote endothelialization of covered stents, drug eluting covered stents have been used^32–34^. Zhang et al.^32^ designed a biofunctional stent covered with dual drug-loaded electrospun fibers to achieve programmed vascular endothelial growth factor and paclitaxel release for promoting stent endothelialization and long-term prevention of instent stenosis in the treatment of cerebral aneurysms, leading to good outcomes. Chu et al.^33^ developed a controlled-release stent covered with a core–shell nanofiber mesh fabricated by emulsion electrospinning for the treatment of aneurysms, and these stents showed good effects in separating the aneurysm dome from blood circulation while inducing neovascularization and keeping long-term patency of the parent artery. Liu et al.^34^ explored a new rosuvastatin calcium- and heparin-loaded poly(l-lactide- co-caprolactone) (PLCL) scaffold for covered stents in the treatment of aneurysms, and these stents demonstrated favorable anticoagulation and pro-endothelialization properties in vitro and in vivo in a rabbit aneurysm model, providing an additional option for treating cerebral aneurysms with covered stents. With the fast development of technology, more and better covered stents will be able to developed for successful treatment of cerebrovascular diseases with endured effects.

The strengths of our study included the serially timely investigation of endothelialization from 20 days up to 10 months after deployment of the stents. Moreover, the percentage of endothelialization had also been compared in different stents, with no significant difference being revealed between different stents. The percentage of endothelialization is gradually increased with time. With time, the endothelialization of the stents became maturer with time. Currently, no studies have been performed to compare the endothelialization in covered stents and bare stents with such a long time (up to 10 months after deployment). Even so, some limitations existed in this study, including a small sample of animals, no test of the blood parameters like creatinine, and a short follow-up period. Moreover, the endothelialization maturation of the stent was not quantified, and no quantitative comparison was made between different stents or between different observing time points except for the extent of stent endothelialization. The quantification of the stent endothelialization maturation needs to be solved with novel techniques so as to make detailed comparison for stent endothelialization on different stents or at different time points in the future. Future studies will have to overcome these issues for better outcomes by including a large animal sample, animal blood test, quantification of the endothelialization, and a longer follow-up duration.

In conclusion, three months after deployment in canines’ arteries, the self-expandable bare and covered stents have mostly been covered by endothelial cells which become maturer over time, whereas the balloon-expandable covered stents do not have complete coverage of endothelial cells at three months, especially for protruding stent struts and areas. Over time, the endothelialization will become mature.

## Data Availability

The datasets generated and/or analysed during the current study are not publicly available because no data have been allowed to make publically available in our hospital but are available from the corresponding author on reasonable request.
